# Comparing endoscopic ultrasound (EUS)-guided fine needle aspiration (FNA) versus fine needle biopsy (FNB) in the diagnosis of solid lesions: study protocol for a randomized controlled trial

**DOI:** 10.1186/s13063-016-1316-2

**Published:** 2016-04-12

**Authors:** Jinlin Wang, Xiaoli Wu, Ping Yin, Qiaozhen Guo, Wei Hou, Yawen Li, Yun Wang, Bin Cheng

**Affiliations:** Department of Gastroenterology and Hepatology, Tongji Hospital, Tongji Medical College, Huazhong University of Science and Technology, Wuhan, 430030 China; School of Public Health, Tongji Medical College, Huazhong University of Science and Technology, Wuhan, 430030 China

**Keywords:** EUS-FNA, EUS-FNB, Slow-pull, Suction, Solid lesions, Diagnostic yield

## Abstract

**Background:**

Linear endoscopic ultrasonography (EUS) allows the visualization, identification, and characterization of the extent of lesions of the gastrointestinal (GI) tract and adjacent structures. EUS-guided fine-needle aspiration (EUS-FNA) facilitates a more accurate diagnosis of mediastinal, intra-abdominal, and pancreatic lesions through the collection of the cytological material under direct visualization. Recent reports suggest that histological samples can be obtained by EUS-FNA with a reverse, bevel-tipped needle (the ProCore needle) to collect the core samples (fine needle biopsy, FNB), thereby adding a new dimension to the diagnostic usefulness of this technique. Certain neoplasms, such as lymphoma and stromal tumors, can be assessed by EUS-FNB to confirm the diagnosis. Here, we aimed to carry out a prospective, multicenter, single-blind, randomized, controlled trial to compare EUS-FNB and EUS-FNA.

**Methods/design:**

A total of 408 patients will be enrolled from five endoscopic centers. Patients will be divided into two groups: (1) group A, which is the EUS regular needle group (EUS-FNA) and (2) group B, which is the EUS ProCore needle group (EUS-FNB). Patients in group A will be examined with a 22G EchoTip Ultra needle, and patients in group B, with a 22G EchoTip ProCore needle. For all included patients, four EUS-guided passes will be made in each lesion. In the first and second pass, a slow-pull suction method of the stylet will be done. The third and fourth pass will use manual suction of 5 cc. The primary objective is to compare the diagnostic yield of malignancy by EUS-FNA versus EUS-FNB.

**Discussion:**

The trial will compare samples obtained by EUS-FNA versus EUS-FNB for the diagnostic yield of solid lesions. The efficacy of these two sampling methods will be assessed on various lesions, which may provide insights into developing practice guidelines for their future indications.

**Trial registration:**

Clinical Trials.gov, NCT02327065.

## Background

Linear endoscopic ultrasonography (EUS) allows the identification of suspected malignancies by creating real-time images of the digestive tract and adjacent lesions [1]. EUS-guided fine needle aspiration (FNA) offers an opportunity for sampling mediastinal, intra-abdominal, and pancreatic lesions under direct visualization [2]. Since its original description in the early 1990s, EUS-FNA has been used to obtain cytological material, thereby contributing to the prompt and accurate diagnosis of clinically suspected lesions [3–6]. The diagnostic accuracy of EUS-FNA is generally high, from 52 % to 94 % [7–10]. Cytopathology plays an important role in improving the diagnostic yield. Notably, distinguishing the inflammatory lesion caused by a reaction and regeneration from well-differentiated neoplasia solely based on cytological evaluation can be difficult. Moreover, certain neoplasms, such as lymphoma and stromal tumors, require histological exams to assess the tissue architecture and cell morphology changes in order to confirm the diagnosis [11]. To overcome this limitation, a new fine needle biopsy (FNB) device has been designed (Cook Endoscopy, Limerick, Ireland). The new ProCore needle was designed with a reverse bevel at the tip to collect a core sample. A multicenter study on 114 patients showed that the application of the ProCore needle (EUS-FNB) led to 85.96 % diagnostic accuracy; 90.2 % sensitivity to malignancy, 99 % specificity, 100 % positive predictive value (PPV), 78.9 % negative predictive value (NPV), and 92.9 % accuracy [11]. Overall, most studies favored EUS-FNB for achieving an adequate histological specimen and high diagnostic yield compared to EUS-FNA [11–16]. However, several studies have suggested that the diagnostic yields of EUS-FNB and EUS-FNA were similar [17–19]. Therefore, the impact of the needle type (ProCore or regular) on the diagnostic yield needs to be further studied. We plan to carry out a randomized controlled trial to compare the diagnostic yield of EUS-FNA versus EUS-FNB and to assess the sample quality obtained by both the slow-pull and the suction approaches.

## Methods/design

The study was approved by The Ethics Committee of Tongji Medical College, Huazhong University of Science and Technology (HUST) (IORG No: IORG0003571). Informed consent will be obtained from the patients or from their closest relatives with authorization.

### Study design

**Fig. 1:**
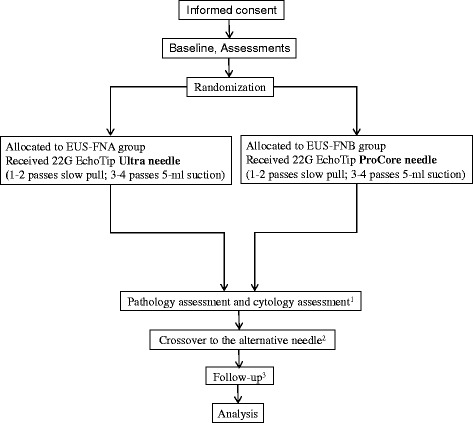
CONSORT flowchart illustrating the randomization and recruitment process in the study. ^1^ Each slide will be assessed by two independent experts. The cytologists and pathologists will follow the protocol to assess the samples and will be blinded as to which technique is used for which specimen. ^2^ If diagnostic failure occurs, the patient will be allowed to crossover to the other group after agreeing to accept a needle biopsy again on the same lesion 1 week later. ^3^ Three follow-up points are scheduled after the biopsy (1-week postoperational follow-up, 12-week follow-up, and 48-week follow-up); thereafter, the follow-ups will be conducted via telephone interviews or outpatient interviews. Once the surgical pathologic results are obtained, we will stop the follow-up

A total of 408 patients from five endoscopic centers in China will be enrolled in this prospective, multicenter, randomized, controlled trial. They will be randomly divided into two groups: (1) group A, which will receive the EUS procedure using the regular needle (EUS-FNA, 22G EchoTip Ultra needle, Cook Endoscopy, Limerick, Ireland) and (2) group B, which will receive the EUS procedure using the ProCore needle (EUS-FNB, 22G EchoTip ProCore needle, Cook Endoscopy, Limerick, Ireland). The recruitment process flowchart is shown in Fig. 1.

### Objectives

The primary objective of this study is to compare EUS-FNA versus EUS-FNB for the diagnostic yield (%) of malignancy [13]. The secondary objectives are to compare EUS-FNA versus EUS-FNB for 1) the overall diagnostic yield (%) from solid lesions; 2) the diagnostic yield (%) from pancreatic and nonpancreatic solid lesions, 3) the crossover diagnostic yield (%), and 4) the sample quality obtained by either the slow-pull or suction approaches, respectively.

### Setting

A total of 408 patients will be recruited from five endoscopic centers, including Tongji Hospital, Tongji Medical College, HUST, Wuhan; Cancer Institute & Hospital, Chinese Academy of Medical Sciences, Peking Union Medical College, Beijing; Fudan University Shanghai Cancer Center, Shanghai Medical College, Fudan University, Shanghai; Sun Yat-sen University Cancer Center, Guangzhou; and the First Affiliated Hospital, College of Medicine, Zhejiang University, Hangzhou. All clinic sites meet the following criteria: (1) at least 48 patients can be recruited per site; (2) at least 2 to 4 trained physician(s) and nurse practitioner(s) per center can participate in the trial; and 3) the site agrees to strictly abide by the study protocol.

### Eligibility

#### Inclusion criteria

Patients who meet the following inclusion criteria will be recruited to the trial:

1. Age > 18 years old
2. Sex: male or female
3. Patients who require endoscopic ultrasound and tissue sampling after imaging examination (MRI, CT, and ultrasonography) that shows either pancreatic, intra-abdominal, mediastinal, or pelvic cavity solid lesions (size > 1 cm)
4. Patients who are willing to be examined in the trial centers
5. Patients who are able to give consent

#### Exclusion criteria

Patients will be excluded from the trial for any of the following reasons:

1. Hemoglobin ≤ 8.0 g/dL
2. Pregnant female
3. Patient has any coagulation disorder (PLT < 50,000/mm^3^, INR > 1.5)
4. History of taking oral anticoagulation agents such as aspirin, warfarin, etc. in past week
5. Experienced acute pancreatitis in past 2 weeks
6. Has cardiorespiratory dysfunction that cannot tolerate the operation
7. Has mental diseases or drug addiction
8. Unable to provide informed consent

### Randomization and blinding

The randomization will be conducted at the study office at the School of Public Health, Tongji Medical College, HUST. A stratified block randomization will be used, and the block size is 8. The patients will be allocated randomly to either group A (EUS-FNA) or group B (EUS-FNB) (1:1).

A data manager, who will not be involved in the data analysis or patient enrollment, will generate the randomization schedule. To ensure allocation concealment, the randomization schedule will be sealed under scratch cards (001–408). The scratch cards will not be opened until the baseline assessment is completed and the patient has consented to participate in the study.

The randomization schedule will not be available to the study recruiters or echoendoscopists until the baseline assessment is completed. Only the study coordinators have access to the randomization schedule. The cytologists and pathologists will be blinded during the entire study.

### Interventions

Patients will be allocated randomly into group A (EUS-FNA, n = 204) or group B (EUS-FNB, n = 204). Group A patients will be examined with the 22G EchoTip Ultra needle, and group B will be examined with the 22G EchoTip ProCore needle.

The procedure will be performed under deep sedation according to the principles of “monitored anesthesia care.” The patients will be anesthetized with intravenous administration of propofol by trained anesthetists. All patients will receive oxygen during the procedures; blood pressure and heart rate will be monitored. Once the lesion is evaluated by EUS, the echoendoscopist will select the shortest pathway, while avoiding blood vessels, to reach the lesion. Under real-time visualization, each lesion will be punctured with four needle passes. To eliminate technical biases, the same procedure for obtaining samples will be applied to each patient undergoing EUS-FNA or EUS-FNB.

### Technique for EUS-FNA and EUS-FNB

**1) The first and second needle passes (slow pull)** [20]: The needle will be advanced into the lesion under real-time EUS visualization. Back and forth movements will be made 20 times within the lesion and will be performed using simultaneous minimal negative pressure by pulling the needle stylet slowly and continuously.

**2) The third and fourth needle passes (5-ml suction)** [18]: After the needle has been advanced into the lesion under real-time EUS visualization, the stylet will be removed. Continuous suction will be applied with a 5-ml syringe, and the needle will be moved back and forth 20 times within the lesion. Suction will be released, and then, the needle will be withdrawn from the lesion.

After biopsy, the samples will be labeled immediately with numbers in the order of the needle pass. Samples will be prepared for histological and cytological examinations. The cytologists and pathologists will always be blinded regarding which technique was used for which specimen.

After the selected lesion has been punctured during the four needle passes, if no core tissue is obtained or if the operators determine the specimens are insufficient according to the results of the macroscopic onsite evaluation (MOSE) [21], the operators will use a proper puncture method to obtain samples as the backup procedure.

If diagnostic failure occurs, the patient will crossover to the other arm after agreeing to accept a needle biopsy again on the same lesion 1 week later with the same method mentioned above.

### Assessment standards

After patient recruitment is completed, all samples will be reassessed by selected cytologists and pathologists from Tongji Hospital, Tongji Medical College, HUST, and from the Cancer Institute & Hospital, Chinese Academy of Medical Sciences, Peking Union Medical College. They will assess the samples following the protocol and remain blinded regarding which technique was used for which specimen. Each sample will be assessed by two independent experts. If the two experts have different judgments, they should discuss it together and make a final decision [7].

#### Histology assessment criteria

The histological assessment will involve the following:

1. Tissue integrity assessment [20, 22]
  Grade A: core tissue (an architecturally intact piece of tissue measuring at least 550 micron in greatest axis, as the diameter of a high-power microscopic field), clearly characterizes the lesion sufficient for diagnosis
  Grade B: core fragments present, tissue does not meet the criteria for architecturally intact histology but can still yield a diagnosis based on cell morphology
  Grade C: no lesion tissue found and cannot yield a diagnosis
2. Blood cell contamination assessment [18]
  Grade A: little blood contamination, minimal surface area (SA) < 25 % of slide
  Grade B: medium blood contamination, 25–50 % of the slide
  Grade C: much blood contamination, SA > 50 % of the slide

#### Cytology assessment criteria

The cytological assessment involves the following:

1. Cellularity assessment [9]
  Grade A: satisfactory, more than four clusters, with a minimum of ten cells in each cluster
  Grade B: adequate, approximately two to four clusters, with a minimum of ten cells in each cluster
  Grade C: unsatisfactory, fewer than two clusters, or no cellular smear
2. Blood cell contamination assessment [18]
  Grade A: little blood contamination, SA < 25 % of slide
  Grade B: medium blood contamination, 25–50 % of slide
  Grade C: much blood contamination, SA > 50 % of the slide

### Collecting data

Table 1 shows the types of data collected and when the data should be collected.

**Table 1:** Study schedule for data collection

Item	Interview 1	Interview 2	Interview 3	Interview 4
	1-0 week before EUS	0-1 week after EUS	12 weeks ± 5 days after EUS	48 weeks ± 5 days after EUS
Informed content	X			
Inclusion criteria	X			
Patient characteristics	X			
Blood routine tests	X	X		
Coagulation routine tests^1^	X			
Blood biochemistry tests^2^	X	X		
Blood tumor markers tests^3^	X		X	X
Complications		X	X	X
Clinical signs^4^	X	X	X	X
Imaging examination	X	X	X	X
Cytology examination		X		X
Pathology examination		X		X
Surgical-pathological examination		X	X	X
Therapies		X	X	X

^1^Coagulation routine tests: prothrombin time (PT), activated partial thromboplastin time (APTT), thrombin time (TT), fibrinogen, INR

^2^Blood biochemistry tests: AST, ALT, BUN, Cr, ALP, lipase, amylase

^3^Blood tumor marker tests: CEA, CA19-9, CA72-4, AFP, SSC, NSE

^4^ Clinical signs include pain, weight loss, cachexia, etc.

### Final diagnosis

The reports will be stratified into four diagnostic categories for the histological and cytological evaluation, including “positive for malignancy,” “suspicious for malignancy,” “atypia,” and “negative for malignancy.” The “positive for malignancy” will be considered if the reports contain words such as “diagnostic for malignancy,” “compatible with carcinoma,” “consistent with adenocarcinoma,” “positive for malignant cells,” “malignant cells present,” or when specified by the exact tumor type [23]. The “positive for malignancy” will not be considered if the reports contain words such as “suspicious for malignancy,” “atypia,” or “negative for malignancy.”

#### Final diagnostic criteria

The final diagnostic criteria [20] include the following:

1. For patients who undergo surgery, the final diagnoses will be based on the definite histological diagnoses from surgical resection specimens.
2. In the absence of surgical pathology, a minimum 48 weeks of clinical follow-up time will be conducted. If the lesion spontaneously resolves or has no sign of deterioration in follow-up imaging studies, the lesion will be considered a benign disease. If the lesion shows enlargement or metastasis, and the patient presents malignant symptoms such as weight loss, anemia, or dies of tumor complications during the follow-up, the lesion will be considered a malignant disease.

### Statistical analysis

#### Sample size

We assumed that the diagnostic yield of malignancy for EUS-FNA was 80 % [23] versus 93 % for EUS-FNB [11, 13]. Because approximately 70 % of patients are expected to be diagnosed with malignancy [9], the sample size should be 342, with a power of 85 % and a two-sided significance level of 5 %. Considering 20 % of patients will be lost to follow-up, the estimated patient number in this trial should be 408.

#### Data analysis

A two-tailed distribution will be used and a *P*-value < 0.05 is considered statistically significant. Continuous variables will be measured as mean (range) and standard deviation using *t* tests or Wilcoxon rank-sum tests. Categorical variables will be measured as the count and percentage using the χ2 test. The diagnostic yield will be described as a proportion using the χ2 test; the blood contamination and cellularity in specimens with slow-pull and suction will be divided into three levels (Grade A, Grade B, and Grade C), and McNemar’s test for correlated proportions will be used. All analyses will be performed with SAS version 9.2.

## Discussion

The role of EUS-FNA in obtaining cytological materials is well-established [24]. However, a recent study suggests the false positive rate of FNA cytology is 5 % to 7 %, which is higher than the originally reported rates of 0 % to 1 % [25, 26]. Moreover, an increased interest in histological tissue sampling under real-time EUS imaging exists because of the advantage in diagnosing certain suspected tumor types, such as neuroendocrine tumor and lymphomas [27]. A standard 19G FNA needle has been used to obtain histological materials and achieved a diagnostic accuracy of 94.5 % [28], although the device is highly associated with technical failures. A Quick-Core needle was used with an overall diagnostic accuracy of 75 % to 84 % [29, 30]. One drawback of the Quick-Core needle is that it is difficult to maneuver, especially for transduodenal biopsy. Recently, a new 22G needle with a reverse bevel at the tip (EUS-FNB) has become available. The ProCore needle design combines the features of an FNA needle with a core biopsy needle [20], which has enhanced flexibility, especially for core tissue collection. Results from a multicenter study on EUS-FNB showed that, overall, the operations were completed in 98.24 % patients, the histological specimens were adequate in 89.47 % patients, and the diagnoses were accurate in 92.9 % patients [11]. EUS-guided tissue acquisition now seems to be more suitable for histological evaluation [13, 20]. Here, we carry out a randomized controlled trial to compare the diagnostic efficiency of EUS-FNB versus EUS-FNA and to assess the sample quality obtained by different types of needles with different methods. To our knowledge, this is the largest cohort study on EUS-FNB so far. The outcome measurements will help us to understand the special indications for each technique and their diagnostic accuracy. In addition, Aadam et al. reported a significant rescue effect of FNA crossover to FNB [13]. We specifically designed our trial to allow the patient to crossover to the other arm if diagnosis is not achieved after the first round of needle biopsy, which will help us draw our conclusions.

The success of EUS-FNA is associated with several factors, including appropriate methods of biopsy, sample preparation, and onsite cytopathological interpretation [31]. The onsite cytopathological interpretation may not always be available during EUS**-**FNA procedures; therefore, appropriate methods of biopsy and sample preparation are very important. Some echoendoscopists use slow-pull, and others use constant suction. Suction with a self-retracting syringe will likely bring more cellularity but also more blood. In this trial, the echoendoscopist will use both methods (slow-pull and constant suction) on each lesion and, hopefully, will be able to draw a clear conclusion on selecting which biopsy method should be used for which procedure. Furthermore, standard procedures and sample preparation methods will be used to process all types of biopsy samples. For example, after the needle has been inserted into the mass, for both FNA and FNB, the needle will be moved back and forth 20 times within the lesion to collect specimens. The specimens will be expelled from a needle in three steps: step 1, which involves pushing the stylet into the needle; step 2, which involves flushing the needle with 0.1 ml saline; and step 3, which involves filling the needle with 2 ml of air.

Some limitations exist in the present study design. For instance, we will not be performing the rapid onsite evaluation (ROSE) in the current trial. ROSE is most frequently used in the United States, and the absence of an onsite cytopathologist has been shown to affect the diagnostic yield by increasing the number of inadequate samples. Although its popularity is growing here in China as well, several alternative strategies, including the macroscopic onsite evaluation (MOSE), have been reported to improve the diagnostic yield [21]. The well-trained endoscopists in our centers will apply MOSE and gross inspection to evaluate the quality of the specimens. Second, although the crossover design is critical, obtaining agreement from the patients for the second biopsy may be difficult because it may increase the risk of complications. Consequently, the number of patients recruited may be reduced, thereby leading to suboptimal conclusions. Third, some patients may not undergo surgery and, hence, will not have any final pathology report. In addition, the possibility exists that contact with the patient will be lost during the 48 weeks of follow-up. To prevent dropout, local research assistants will be recruited to help complete the patient registration information (record at least two phone numbers) and ensure that appointments are being made for follow-up assessments.

### Trial status

Patient enrollment began on December 20, 2014 and completion is expected by December 31, 2015. At present (December 5, 2015), 372 patients have been enrolled in the study.

## Abbreviations

AFP: Alpha fetal protein
ALP: Alkaline phosphatase
ALT: Alanine aminotransferase
APTT: Activated partial thromboplastin time
AST: Aspartate aminotransferase
BUN: Blood urea nitrogen
CA19-9: Carbohydrate antigen 19-9
CA72-4: Carbohydrate antigen72-4
CEA: Carcinoembryonic antigen
Cr: Creatinine
EUS: Endoscopic ultrasonography
EUS-FNA: Endoscopic ultrasound-guided fine-needle aspiration
EUS-FNB: Endoscopic ultrasound-guided fine-needle biopsy
FNA: Fine needle aspiration
INR: International normalized ratio
MOSE: Macroscopic onsite evaluation
NPV: Negative predictive value
NSE: Neuron specific enolase
PPV: Positive predictive value
PT: Prothrombin time
ROSE: Rapid onsite evaluation
SA: Surface area
SAS: Statistical Analysis System
SSC: Squamous cell carcinoma antigen
TT: Thrombin time
